# Intrathoracic negative pressure therapy for pleural empyema using an open-pore drainage film

**DOI:** 10.1007/s00104-023-01827-8

**Published:** 2023-03-15

**Authors:** V. Betz, V. van Ackeren, E. Scharsack, B. Stark, C. T. Müller, G. Loske

**Affiliations:** Marienkrankenhaus: Katholisches Marienkrankenhaus GmbH, Hamburg, Germany

**Keywords:** Bacterial infection, Thoracotomy, Drainage, Lung, Video-assisted thoracic surgery

## Abstract

**Background:**

We report our initial experience with intrathoracic negative pressure therapy (ITNPT) in the stage-adjusted treatment of pleural empyema (PE) based on a case series.

**Materials and methods:**

ITNPT represents a further development for intrathoracic use. After thoracic surgical open debridement, an intrathoracic negative pressure dressing was inserted. The drainage elements were a thin open-pore double-layer drainage film (OF) with open-pore polyurethane foams (PUF). Only the OF was placed in direct contact with the lung parenchyma. Negative pressure was generated using an electronic pump (continuous suction, −75 mm Hg). In revision thoracotomies, ITNPT was stopped or continued depending on local findings.

**Results:**

In total, 31 patients with stage II and III pleural empyema underwent ITNPT, which was administered during the primary procedure (*n* = 17) or at revision (*n* = 14). Treatment duration was a mean of 10 days (2–18 days) with a mean change interval of 4 days (2–6 days). Intrathoracic negative pressure dressings were applied a mean of 3.5 (1–6) times. The empyema cavity continuously reduced in size and was cleansed by the suction. The OF has a minimum intrinsic volume with maximum absorption surface. Once negative pressure is established, there is no intrathoracic dead volume and the parenchyma can expand. The protective material properties of OF make ITNPT suitable for the treatment of pleural empyema. Targeted local intrathoracic drainage of the septic focus is a possible adjunct to surgery. The surgical dressings must be changed repeatedly. The method is suitable for the treatment of complex stage II and III pleural empyemas.

**Conclusion:**

The OF can be used as an intrathoracic drainage element for ITNPT in pleural empyema. This new application option expands the range of indications for negative pressure therapy.

## Introduction

Pleural empyema (PE) is a serious condition that often requires thoracic surgical treatment. The incidence is increasing worldwide with a mortality of up to 20% [5, 24]. The condition is a bacterial infection of the pleural space involving both pleural layers.

Pleural empyema can be parapneumonic, postoperative after lung surgery, or may occur in other diseases originating in the lungs, such as bronchiectasis [6, 21, 22]. It can also be caused by extrapleural bacterial contamination, e.g., from the abdominal cavity.

According to the American Thoracic Society, PE is divided into three stages depending on the disease course and on the stage of the disease [26, 28].

Treatment should center on controlling the focus of infection, relieving secretions and putrid matter, and re-expanding lung tissue and restoring physiological respiratory chest movements [23].

Supportive parenteral antibiotics are given at all stages, depending on the cause of the infection (community-acquired, nosocomial; [9, 23]).

*In Stage I (exudative phase),* when there is a predominantly sterile effusion without pronounced septal formation and with pleural thickening, treatment takes the form of a thoracic drainage with initially empirical and then targeted antibiotic therapy [1, 8, 25, 26].

*Stage II (fibrino-purulent phase)* is characterized by a thickening of secretions, with the formation of thick fibrin coatings and membranes as well as pus formation in the pleural cavity with a risk of sepsis [1, 25, 29].

For therapy, debridement and, if necessary, decortication are performed, preferably by means of video-assisted thoracoscopy [8, 24, 26].

In *stage III (organizational phase),* PE can develop into a chronic form with adhesions between the pleural layers, rind formation, and tethering of lung tissue [1, 25, 29]. For therapy, a thoracotomy with open surgical decortication is required [10, 23, 26, 28].

In addition, a thoracoscopic approach using video-assisted thoracoscopy (VATS) has been attempted in stage III in recent years [11, 23].

Negative pressure therapy is widely used in the treatment of superficial or deep wounds that are healing by secondary intention and are located in areas of the body that are difficult to access. Open-pore polyurethane foams (PUF) are inserted into the wound and sealed with an adhesive film. Negative pressure is created by connecting a hose to an electronic pump. On the body surface, negative pressure therapy is also used for temporary abdominal wall closure and for larger tissue defects, e.g., of the abdominal or thoracic wall [3, 4].

Another important indication for negative pressure therapy is its intra-abdominal application for the treatment of peritonitis. A more recent development is the intracorporeal application of endoscopic negative pressure therapy in the management of postoperative complications relating to anastomotic insufficiencies of the upper and lower gastrointestinal tract. [13, 15–19].

Based on a case series of 31 patients, we present our initial experiences of treating PE with intrathoracic negative pressure therapy (ITNPT) using an open-pore drainage film.

## Methods

### Patients

Inclusion criteria were patients with pleural empyema in advanced stages II and III as a result of pneumonia (*n* = 14), recurrent empyema (*n* = 2), or lung abscess (*n* = 5) confirmed by computer tomography or per puncture, as well as complicated courses after initial surgery, such as after previous pneumonectomy (*n* = 5), after partial lung resection in the context of malignancy (*n* = 2), and after esophageal perforation or resection (*n* = 1); two patients had empyema necessitatis (*n* = 2).

The patients were informed verbally and in writing before the surgical procedures regarding the possible need for an intraoperative ITNPT.

### Technical requirements

Within a few years, negative pressure therapy has become established as an important therapeutic measure [3, 17]. In this context, ITNPT represents a further development for intrathoracic use.

The technical principle of negative pressure therapy (NPT) is similar in all areas of application. The technical requirements are:

- A compartment in which a negative pressure can be established
- A negative-pressure-generating system, e.g., an electronic pump, that is connected to it via hoses
- An open-pore drainage element (DE) capable of providing suction

Along the surface of the open-pored DE, therapeutic negative pressure is applied to the surface to be treated.

### Compartment

Negative pressure therapy requires a sealed, airtight compartment. For NPT on the body surface, this is created using occlusive films. When used in the abdominal cavity, it must be hermetically sealed after surgical opening using occlusive dressings and/or surgical sutures to establish negative pressure.

Similarly, this also applies to ITNPT. Here, too, the wound is closed after the thoracic surgery by sutures or using an occlusive dressing. A special prerequisite is that the pleural cavity has a physiological level of negative pressure in order for the lung parenchyma to expand.

### Negative-pressure-generating system

An electronic pump was used to generate negative pressure for ITNPT, which was originally developed for body-surface therapy, but is also used in abdominal NPT and endoscopic NPT (ACTIV.A.C.™ Therapy System, KCI USA, Inc., San Antonio, TX, USA).

**Fig. 1:**
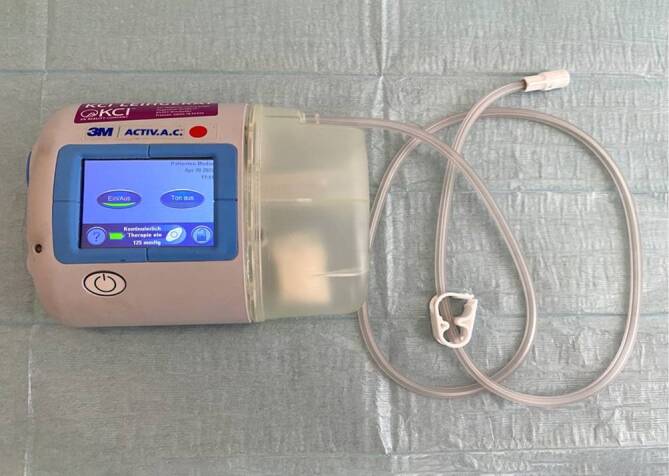
Pump system with canister and drainage connection hose

A therapeutic negative pressure of −75 mm Hg and continuous suction mode were applied as standard (Fig. 1).

### Open-pore drainage elements

Two different drainage materials were used to provide suction for ITNPT:

- Open-pore double-layer drainage film (OF)
- Open-pore polyurethane foam (PUF)

Both share the physical property of open pores. All the pores in the materials are in liquid- and gas-conducting contact with each other. When negative pressure is applied to one area of the material, it is transmitted to the entire surface and from there to the wound area. Even if some of the pores become blocked, suction is maintained via other communicating openings. The two materials we used differ in their physical properties.

### Open-pore polyurethane foam

The foam body of the open-cell, hydrophobic PUF (V.A.C. GRANUFOAM™ DRESSING, KCI USA, Inc.) has a relatively large volume, which only partially collapses under suction.

The uneven pore openings (400–600 µm in size) are densely adjacent and merge with each other [27]. Depending on the size of the foam surface, pore size, and consistency of the wound surface, they can be suctioned very firmly to the wound bed and accordingly adhere tightly.

The PUF has strong debriding properties on the wound surface and can be used on body surface wounds for wound cleansing and conditioning. A contraindication is direct application to the peritoneum and blood vessels.

**Fig. 2:**
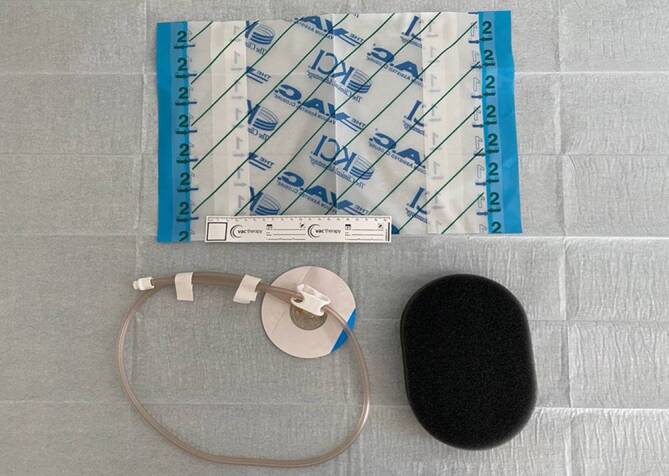
Adhesive film and trackpad for pump connection, polyurethane foam for suction transfer to the thoracic negative pressure film

Due to the possibility of internal wound contact, there is a significant risk of erosions resulting from the material and the suction. The development of transmural intestinal fistulas and bleeding have been described as severe complications ([2, 14]; Fig. 2).

### Open-pore double-layer drainage film

The OF (Suprasorb® CNP Drainage Film, Lohmann & Rauscher International GmbH & Co. KG, Rengsdorf, Germany) is a very thin drainage material measuring 77 × 60 cm or 25 × 20 cm. It is composed of two perforated membranes separated by a cavity that does not collapse under suction.

**Fig. 3:**
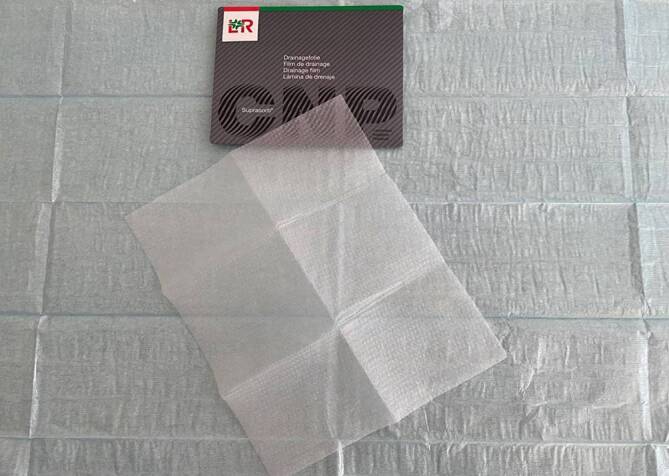
Open-pore double-layer drainage film placed intrathoracically directly on the lung parenchyma

**Fig. 4:**
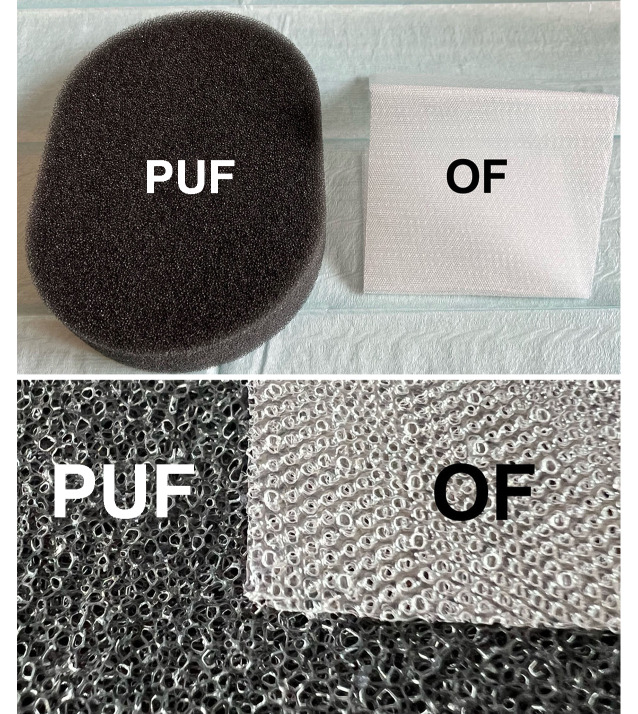
Comparison of the surface structure of polyurethane foam (*PUF*) and open-pore double-layer drainage film (*OF*)

This enables open-pore drainage of liquids and gases along the film membranes, through them, and in the cavity between them. The negative pressure is generated over the entire surface of the drainage film. The film does not collapse under suction. It has a minimal volume with a very large absorption area. Another difference when compared with the PUF is the spacing of the pores. In the OF, uniformly shaped pores are arranged at a regular and close, bridge-like distance from one another. It does not adhere as firmly to the tissue as the PUF. The debridement effect is weaker but gentler than with the PUF. The risk of erosion in vulnerable structures is lower. In contrast to the PUF, the OF can be placed directly on the peritoneum. Due to its flexible material properties, it can be easily molded onto the organs (Figs. 3 and 4).

The PUF and the OF can be used in a complementary way. The PUF can be encased within the OF (OFPUF). The OFPUF has the gentle surface properties of the OF.

Depending on local intrathoracic findings, only the OF and the OFPUF were used intrathoracically in our patients: For non-removable fibrin coatings, the OF was applied directly to the lung parenchyma; for localized fluid infiltration, we inserted an OFPUF. In superficial extrathoracic soft tissue, we used PUF, OF, and OFPUF.

### Operative procedure and negative pressure dressing application

Surgically, after anterolateral thoracotomy and after entering the thoracic cavity, both pleural layers were typically decorticated, and encapsulated pus collections and fibrinous abscess membranes were removed.

After extensive irrigation and suction of the irrigation fluid, the OF was then cut to the required size, adapted to the wound surface, and inserted into the pleural cavity. An attempt was made to loosely enclose the entire affected and inflamed lung parenchyma with the large-area OF, so that the parenchyma could expand while remaining encased in the film. The OF was thus in the pleural cavity and was released into the chest wound. At the opening of the thoracic wound, the OF was brought into direct contact with a PUF adapted to the wound opening.

The wound edges were covered with a 3M™ Cavilon™ skin protection film. The thoracic wound was not closed surgically, but sealed with an occlusive film, and the trackpad was then placed over this to create a vacuum.

Once it was connected to the pump, a negative pressure of −75 mm Hg was applied to the dressing. The PUF and the intrathoracic OF were in a direct negative pressure conducting connection via material contact.

**Fig. 5:**
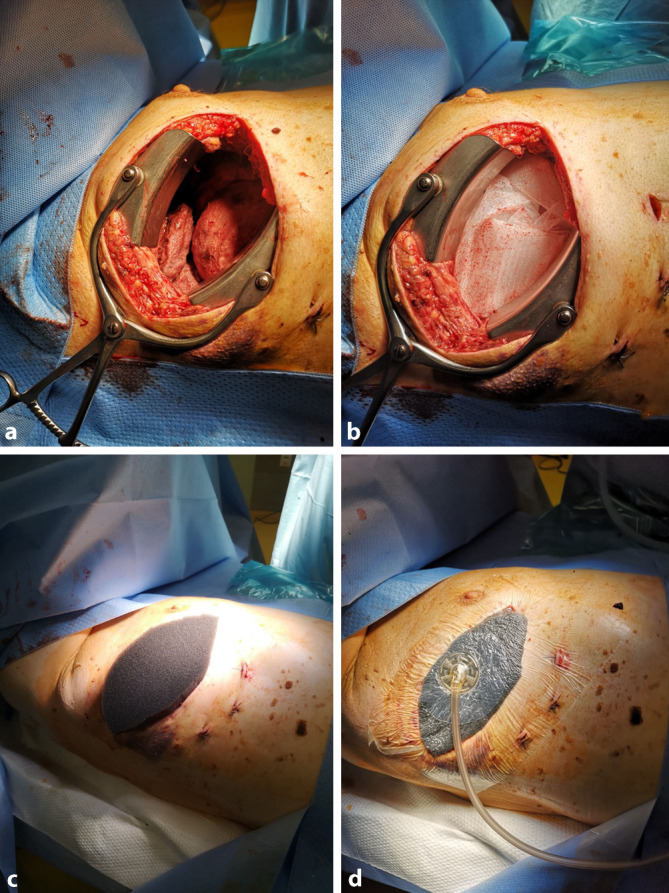
**a**–**d** Surgical application of the negative pressure film dressing. **a** Open thoracic cavity after debridement of the pleural empyema; **b** film insertion in the wound area; **c** polyurethane foam insertion; **d** occlusion of the wound with adhesive film, suction mediated via trackpad

The negative pressure was directed intrathoracically via the contact suction of the open-pore materials (Fig. 5).

### Dressing change interval

Planned dressing changes after the start of negative pressure therapy were performed every 2–6 days depending on the severity of both the intrathoracic and the clinical findings.

To this end, the inserted negative pressure dressing system was removed under surgical conditions, new swabs were taken from the pleural cavity, the pleural cavity was extensively irrigated, and the irrigation fluid was aspirated. Depending on the wound condition, the negative pressure dressing was reapplied in the manner described.

## Results

From March 2017 to October 2021, 216 patients with pleural empyema were treated in the Department of General, Visceral, Thoracic and Vascular Surgery at Marienkrankenhaus Hamburg gGmbH. Overall, 31 patients (14%) underwent surgical treatment with ITNPT (Table 1).

The patients were referred to us from other specialist departments and hospitals for surgical treatment after primary therapy in those departments, and some patients had an extensive medical history. A total of 20 male and 11 female patients aged 31–85 years were treated.

Intraoperatively, advanced stage II and III PE were present, as well as complicated courses after initial surgery.

Overall, 18 patients had undergone prior treatment; of whom five had received a thoracic drainage and 13 had undergone prior surgery.

We performed an anterolateral thoracotomy (THT) as initial surgery on 30, two of whom underwent conversion thoracotomy after initial VATS; one patient initially underwent VATS with a subsequent need for revision thoracotomy.

In 17 patients, an OFPUF was inserted at the time of the initial surgery because of the presence of severe findings; in 14 patients, the OFPUF was inserted because of findings that did not improve in the course of the procedure or as an alternative treatment method.

The remaining patients required negative pressure therapy as part of revisions performed for other reasons. The reasons for revision were hemothorax (*n* = 10), chylothorax (*n* = 1), recurrent empyema (*n* = 13), persistent parenchymal fistulas with seropneumothorax, and in some cases skin emphysema (*n* = 10), intraparenchymal abscess requiring wedge resection (*n* = 1), wound healing disorders of the thoracic wall (*n* = 9) with thoracic wall hematoma or abscess, sepsis (*n* = 2), and spontaneous esophageal perforation with fistula (*n* = 1).

In four patients a leak occurred in the negative pressure system requiring revision.

Five patients underwent omentoplasty during follow-up; four patients required thoracoplasty during follow-up. One patient required a thoracostomy. Five patients underwent tracheostomy as part of long-term ventilation.

With a median of 3.5 VAC procedures (range: 1–6 procedures) and a median VAC change interval of 4 days (range: 2–6 days), the median ITNPT treatment duration was 10 days (range: 2–18 days). The median number of total surgical procedures required was 8 (range: 2–16 procedures).

As a result of the continuous suction, a fine-pore regular suction pattern could be observed on the lung surface through the surface structure. The parenchyma became softer on digital palpation and more expansile on air insufflation.

The wound surfaces appeared cleaner with each dressing change. The empyema cavity visibly shrank during treatment. In addition, incipient granulation of both the wound cavity and the subcutaneous tissue could be observed due to the continuous suction.

The negative pressure dressings were well manageable in terms of skin maceration; no inflammatory changes were observed as a result of the dressing application.

Definitive thoracic wall closure was performed for 26 patients after appropriate improvement of local findings following the application of NPT therapy using a double-layer, open-pore film in the conventional manner under surgical conditions by layered adaptation of the thoracic wall layers, followed by the insertion of intrapleural chest tubes (a.k.a. Bülau drains, two pieces, straight and curved, 28 ch. each).

### Complications

A septic splenic infarct requiring splenectomy (*n* = 1), an intraoperative diaphragmatic tear (*n* = 1), hydronephrosis requiring double‑J stent insertion (*n* = 1), and a lower leg stump abscess requiring drainage that occurred in the context of sepsis (*n* = 1) were additional complications requiring treatment that were observed during therapy but which were not directly related to ITNPT.

Four patients died in the course of inpatient treatment; all deaths were caused by septic shock in combination with acute renal failure (*n* = 2), septic cardiomyopathy with secondary septicemia (*n* = 2), and multiple organ failure (*n* = 1; Table 1).

**Table 1:** Patient overview with treatment data

Patient number	Age (years)	Sex	Disease	Former treatments (yes/no)	Date of primary surgical procedure	Primary surgical procedure	Number of VAC procedures	Change intervals (days)	Overall duration of VAC treatment (days)	Overall number of surgical procedures	Overall duration of surgical treatment (days)	Length of stay in hospital, surgical ward (days)	Complications	Tracheostomy	Death (cause)
1	57	m	Parapneumonic empyema right side with abscess middle lobe	Yes (drainage)	30.03.2017	Thoracotomy/decortication right side	3	4	12	16	62	109	Hemothorax, fistula formation, thoracoplasty, vacuum-assisted closure (VAC) leakage	Yes	No
2	74	m	Chronic empyema left side after pneumonectomy for lung carcinoma 2013	Yes (pneumonectomy left side for lung carcinoma 2013)	27.06.2017	Re-thoracotomy left side with CNP film insertion	1	3	3	2	4	25	Omentoplasty	No	No
3	74	m	Chronic empyema left side after pneumonectomy for lung carcinoma 2013	Yes (pneumonectomy left side for lung carcinoma 2013)	04.12.2017	Thoracotomy, partial thoracoplasty left side with CNP film insertion	2	4	8	3	8	20	Recurrent empyema, thoracoplasty with latissimus dorsi transposition flap	No	No
4	74	m	Chronic empyema left side after pneumonectomy for lung carcinoma 2013	Yes (pneumonectomy left side for lung carcinoma 2013)	04.01.2018	Re-thoracotomy left side, decortication, CNP film insertion	3	5	15	4	14	20	Extension of thoracoplasty	No	No
5	74	m	Chronic empyema left side after pneumonectomy for lung carcinoma 2013	Yes (pneumonectomy left side for lung carcinoma 2013)	31.03.2018	Re-thoracotomy, CNP film insertion	2	6	12	3	11	33	Thoracostomy	No	No
6	55	m	Parapneumonic empyema right side	No	25.09.2017	Thoracotomy/decortication right side	1	2	2	4	3	15	Hemothorax requiring revision	No	Yes (septic shock, acute renal failure, acute cardiomyopathy)
7	55	m	Parapneumonic empyema both sides with abscess	No	30.03.2018	Thoracotomy/abscess drainage both sides	1	4	4	5	35	112	Fistula formation, omentoplasty	Yes	No
8	64	f	Postpneumonic abscess superior lobe left side	No	14.04.2018	Thoracotomy/superior lobe resection left side	1	6	6	3	16	77	Septic splenic infarction; splenectomy	Yes	Yes (septic dissemination, septic cardiomyopathy, septic shock)
9	53	m	Empyema necessitatis left side	No	25.05.2018	Thoracotomy/decortication with CNP film insertion left side	1	4	4	2	5	23	None	No	No
10	45	m	Empyema left side after resection of inferior lobe for destroyed lung/abscessing pneumonia	No	19.11.2018	Thoracotomy/inferior lobe resection left side	3	5	15	12	38	162	Hemothorax, recurrent empyema, wound healing disorder, omentoplasty	No	No
11	85	f	Parapneumonic empyema stage III right side	Yes (drainage)	21.11.2018	Video-assisted thoracoscopy/conventional thoracotomy/decortication right side	1	3	3	6	21	72	Hemothorax, recurrent empyema	No	No
12	65	m	Chronic cutaneous fistula formation/serofibrothorax left side after hyperthermic intrathoracic chemotherapy (HITOC)/pneumonectomy	Yes (surgery HITOC/pneumonectomy left side 09/18)	15.12.2018	Re-thoracotomy, fistula excision, debridement serofibrothorax, CNP film insertion left side	6	3	18	7	18	82	Hemothorax, empyema, wound healing disorder, VAC leakage	No	No
13	66	m	Empyema and abscess inferior lobe left side	Yes (thoracotomy left side/decortication/lung abscess drainage 10.05.19)	06.06.2019	Thoracotomy/decortication/abscess drainage left side	2	4	8	7	40	73	Recurrent empyema, seropneumothorax, fistula formation, omentoplasty	Yes	Yes (septic shock, acute renal failure)
14	31	m	Empyema and abscess superior lobe right side	Yes (drainage)	29.01.2020	Video-assisted thoracoscopy/wedge resection/decortication; thoracotomy right side in the course	3	3	9	7	20	61	Recurrent empyema, fistula formation, lung abscess, hemothorax	No	No
15	82	f	Parapneumonic empyema right side	No	24.02.2020	Thoracotomy/decortication right side	1	4	4	3	15	30	Hemothorax	No	No
16	57	f	Recurrent empyema left side	Yes (thoracotomy/decortication left side 04/20)	12.06.2020	Thoracotomy/decortication with CNP film insertion left side	1	3	3	2	4	13	None	No	No
17	79	m	Empyema right side after superior lobe resection right side for lung carcinoma	No	22.07.2020	Thoracotomy/superior lobe and middle lobe resection right side	1	4	4	5	20	50	Hemothorax requiring revision, fistula formation, effusion, skin emphysema	No	No
18	62	f	Recurrent empyema and abscess inferior lobe right side	Yes (surgery for empyema externally)	06.10.2020	Thoracotomy/decortication right side	1	2	2	4	10	27	VAC leakage	No	No
19	75	m	Parapneumonic empyema left side	No	26.10.2020	Thoracotomy/decortication left side	1	3	3	6	21	38	Recurrent empyema, thoracic wall abscess	No	No
20	55	m	Empyema stage III and abscess inferior lobe right side	No	02.11.2020	Thoracotomy/decortication with CNP film insertion right side	1	2	2	5	12	103	Hemothorax requiring revision, recurrent empyema	No	No
21	78	m	Parapneumonic empyema right side	Yes (drainage)	13.11.2020	Thoracotomy/decortication with CNP film insertion right side	2	3	6	4	26	72	Intraoperative diaphragm rupture, persistent empyema, chylothorax, thoracoplasty	No	No
22	59	m	Empyema with bronchial stump necrosis after resection of superior lobe left side for lung carcinoma	Yes (video-assisted thoracoscopy wedge resection 08/20; superior lobe resection left side 10/20, computed tomography-guided puncture)	23.11.2020	Thoracotomy/bronchus revision resection/CNP film insertion)	2	4	8	3	8	90	Fistula formation, seropneumothorax, recurrent empyema, omentoplasty	No	No
23	70	f	Empyema left side after esophageal perforation following minimally invasive fundoplication	Yes (minimally invasive fundoplication externally 03/21, laparoscopy, Endosponge)	18.03.2021	Thoracotomy/decortication left side	1	4	4	7	79	99	Recurrent empyema, wound infection, omentoplasty with secondary hemorrhage requiring revision, esophagectomy	Yes	No
24	68	f	Empyema stage III and abscess inferior lobe left side	Yes (drainage)	21.06.2021	Thoracotomy/wedge resection/decortication with CNP film insertion left side	2	4	8	5	23	28	Septic shock, thoracic wall hematoma	No	No
25	64	f	Parapneumonic empyema left side	No	24.06.2021	Thoracotomy/decortication with CNP film insertion left side	2	4	8	3	8	24	Hydronephrosis, ureteral double‑J stent insertion	No	No
26	69	f	Parapneumonic empyema right side	No	06.07.2021	Video-assisted thoracoscopy/conventional thoracotomy/decortication right side	5	3	15	8	31	44	Esophageal fistula, recurrent empyema, wound healing disorder, VAC leakage	No	No
27	70	f	Parapneumonic empyema left side	No	12.07.2021	Thoracotomy/decortication with CNP film insertion left side	1	4	4	2	4	8	Wound healing disorder	No	No
28	74	m	Parapneumonic empyema right side	Yes (esophagus resection externally 15.06.21)	16.07.2021	Thoracotomy/wedge resection/decortication with CNP film insertion right side	2	4	8	3	7	16	Fistula formation, wound healing disorder, sepsis	No	Yes (septic shock, multiple organ failure)
29	85	f	Parapneumonic empyema left side	Yes (video-assisted thoracoscopy/decortication left side 08/21)	12.10.2021	Re-thoracotomy left side with CNP film insertion	1	3	3	2	3	10	Recurrent empyema	No	No
30	32	m	Postpneumonic empyema stage II right side	Yes (video-assisted thoracoscopy/conventional thoracotomy/decortication right side 09/21)	19.10.2021	Re-thoracotomy right side, decortication, CNP film insertion	1	3	3	2	3	17	Wound healing disorder	No	No
31	42	m	Empyema necessitatis left side	No	12.10.2021	Thoracotomy/decortication with CNP film insertion left side	2	4	8	7	17	33	Hemothorax requiring revision, abscess left foot requiring revision	No	No

## Discussion

Therapy of PE is based on the disease stage in accordance with the guidelines of the American Thoracic Society [26, 28].

Ideally, the first procedure should be able to resolve the situation once and for all. However, revision procedures are often necessary to remove fibrin and rind tissue that has formed and to prevent tethering of the lung.

When using negative pressure therapy with PUFs, these must not be applied directly to vulnerable tissue, as the adhesion of the foam can cause tissue injuries. In the area of the thorax, negative pressure therapy has therefore mainly been used for external wound treatment on the body surface.

Few reports are available on the intrathoracic use of negative pressure therapy for pleural empyema or lung abscesses [7, 12, 20–22, 28, 30]. In such situations, PUFs with an underlying protective layer, e.g., a micropore silicone film (Mepithel), were used [7, 20].

Based on our many years of experience with OF in endoscopic and intra-abdominal applications, with this clinical, retrospective, non-randomized observational study we present the first case series on the intrathoracic application of negative pressure therapy using a double-layer, OF as a new therapeutic approach. We show that by using this alternative drainage material, negative pressure therapy can also be used in the thoracic cavity as a new therapeutic approach to the treatment of pleural empyema.

The OF, which is applied in direct contact with intrathoracic organs, was originally designed for use in the abdominal cavity. Unlike the PUF, the special material properties of the OF allow it to be applied directly to potentially vulnerable tissue for the application of negative pressure, e.g., in the intra-abdominal treatment of peritoneal organs. There is no need to apply a further protective layer.

The thin OF can be flexibly molded to the lung parenchyma. It allows for expansion with respiration. One clear advantage of the OF is its minimal intrinsic volume and maximum absorption surface. The tiny cavity between the two film sheets does not collapse on suction. Negative pressure is generated over the entire surface of the drainage film. Since the double-layer membrane is very thin, there is almost no dead space volume due to the inserted material after suction is established. The lung tissue can expand fully. At the same time, the empyema cavity shrinks and cleans itself, minimizing the remaining empyema cavity.

A major difference when compared with the PUF is the spacing of the pores. In the OF, uniformly shaped pores are arranged at a regular and close, bridge-like distance from one another. This material therefore adheres less firmly to the material under suction than a PUF. The risk of erosion in vulnerable structures is lower than with a PUF. A PUF can adhere extremely tightly, especially to a wound bed that is already granulating. This property means that vulnerable tissue can be injured and eroded if treatment is prolonged. Direct placement of a PUF on peritoneal organs or vessels is therefore not admissible. There is a risk of bleeding and fistula formation. In our study, we demonstrate that direct placement of the OF with negative pressure is possible even on pleural tissue. Although fistula formation remains a possibility, it was not observed in our case series. In all cases, the OF could be removed gently and without causing injury to the pleura.

In particular, we know from our extensive clinical experience in endoscopic and intra-abdominal negative pressure therapy that the direct debridement effect of the OF is weaker than with the PUF. In the case of intrathoracic application, we observed that the internal wounds were continuously cleansed after surgical debridement with the OF dressing. The tissue in contact with the film has a flat, slightly nodular granulation surface, which disappears completely once treatment has been finalized.

For the sake of completeness, it should be noted that there is also a white polyvinyl alcohol sponge material available that can be used on vulnerable tissues (V.A.C. WHITEFOAM dressing, KCI USA, Inc.). We have no experience in using this material intrathoracically.

Negative pressure therapy is capable of providing optimal and active fluid drainage. Infectious secretions are eliminated by the suction effect, the bacterial load is reduced, inflammatory tissue edema is drained locally over a wide area, and the local perfusion is improved. A clinical marker of this is that the lung parenchyma becomes progressively softer on palpation and more expansile during the course of treatment, which is beneficial for lung ventilation and perfusion.

In line with our experience with abdominal and endoscopic negative pressure therapy, we have chosen a continuous suction of −75 mm Hg as our standard negative pressure. Moderate pressure settings may be equally effective. This question could be investigated in further studies.

The ratio of the total revision procedures to the number of 31 patients seems high at first. However, it is important to bear in mind that this is a patient population in which a complicated course was already present or was to be expected due to the severity of the PE. The revision procedures were pathognomonic for the clinical presentation of pleural empyema and its varying severity in the patient population.

The patients were referred to us from other specialist departments and hospitals after primary therapy in those departments and some patients had an extensive medical history with advanced stages.

Some of the revision procedures were necessary due to serious complications such as hemothorax, chylothorax, parenchymal fistula, cutaneous emphysema, intraparenchymal abscesses, wound healing disorders of the thoracic wall with thoracic wall hematoma or abscess, spontaneous esophageal perforation, and sepsis. These complications were not related to negative pressure therapy, but occurred before the start of ITNPT and in some cases were the indication for treatment.

Due to the selected patient population having advanced empyema at the time of indication, the OF was inserted using an open surgical approach.

In our opinion, in none of the cases discussed would a stage 3 VATS have been sufficient due to severe rind formation; sufficient decortication would not have been achievable with minimally invasive instruments in these cases. Therefore, based on the experience gained in our department, a VATS approach did not seem to be sufficiently promising in these cases, since more revision procedures are often required in advanced stages of empyema after VATS.

A thoracoscopic approach would possibly reduce the length of hospitalization; however, the disadvantage would be a more difficult insertion and a possibly more difficult and incomplete expansion of the OF due to the restricted intraoperative overview compared to the open approach.

Ultimately, one third of the cases still required omentoplasty or thoracoplasty. In these patients, due to pre-existing intraparenchymal abscesses with parenchymal shrinkage, after necessary suturing for persistent parenchymal fistulas and after, e.g., lobectomy, substance defects occurred that made it necessary over time to fill the pleural cavity with omentum majus or, if the substance was insufficient, to perform a thoracoplasty. This is again due to the severity of the original empyema and should not be regarded as a shortcoming of negative pressure therapy.

Except for one case in which a thoracostomy was necessary, the empyema was cured during the treatment. The mortality rate was 12.9% (4 out of 31 patients).

It should be noted that the surgical approach we have chosen results in an increased rate of planned revision procedures simply because of the need for dressing changes. This could legitimately be considered a limitation of the method. It is conceivable that in the future, with further development of the method and novel, adapted drainage materials, minimally invasive techniques could also be used in suitable cases.

### Study limitations

The limitations of the present study are the retrospective analysis and the heterogeneous patient population. The largely standardized procedure consists exclusively of the intrathoracic use of the OF. Wound treatment and dressing application must always be adapted and optimized to the individual circumstances in order to be successful. However, this degree of flexibility is common to all applications of negative pressure therapy. The new drainage materials significantly increase the range of therapeutic possibilities. There are currently no drainage products or pumps approved for intrathoracic therapy.

The need to apply dressings under surgical conditions and anesthesia could also be considered a disadvantage. In principle, this procedure is similar to re-laparotomy in abdominal surgery.

## Summary

In this study, we present intrathoracic negative pressure therapy (ITNPT) as a novel adjunctive treatment option in the surgical management of complicated stage II and III pleural empyema and in complicated courses after initial surgery. An innovative drainage material consisting of a thin, double-layered, open-pore drainage film was used together with conventional, open-pore polyurethane foams originally developed for intra-abdominal negative pressure therapy. Thanks to its surface texture, the drainage film is also suitable for intrathoracic negative pressure therapy applications where there is contact with the lung tissue. This involves the adaptation of negative pressure therapy for intrathoracic use in complicated pleural empyema. Further studies are needed to evaluate the future value of this treatment.

## Abbreviations

DE: Drainage element
ITNPT: Intrathoracic negative pressure therapy
NPT: Negative pressure therapy
OF: Open-pore double-layer drainage film
OFPUF: PUF encased in OF
PE: Pleural empyema
PUF: Polyurethane foam
THT: Thoracotomy
VATS: Video-assisted thoracoscopy

## Declarations

For this article no studies with human participants or animals were performed by any of the authors. All studies mentioned were in accordance with the ethical standards indicated in each case.

## References

[1] Adams O, Ankermann T, Baumann U, Brinkmann F, Bruns R, Dahlheim , Ewig S, Forster J, Hofmann G, Kemen C, Lück C, Nadal D, Nüßlein T, Regamey N, Riedler J, Schmidt S, Schwerk N, Seidenberg J, Tenenbaum T, Trapp S, van der Linden M. S2k-Leitlinie Management der ambulant erworbenen Pneumonie bei Kindern und Jugendlichen (pädiatrische ambulant erworbene Pneumonie, pCAP). AWMF-Register-Nr. 048/030. 10.1055/a-1139-5132 32823360

[2] Andrade D, Wirth U, Renz B, Andrassy J, Werner J (2017). Vakuum-assistierter Wundverschluss einer abdominellen Wunde mit enteroathmosphärischen Fistel.

[3] Beltzer C, Eisenächer A, Badendieck S, Doll D, Küper M, Lenz S, Krapohl BD (2016). Retrospective analysis of a VACM (vacuum-assisted closure and mesh-mediated fascial traction) treatment manual for temporary abdominal wall closure—results of 58 consecutive patients. Reconstr Surg.

[4] Bonnet F, Pavy B, Beaudoin S, Dubousset J, Mitrofanoff M (2007). Scand J Plast Reconstr Hand Surg.

[5] Cargill TN, Hassan M, Corcoran JP (2019). A systematic review of comorbidities and outcomes of adult patients with pleural infection. Eur Respir J.

[6] Deschamps C, Allen MS, Miller DL, Nichols FC, Pairolero PC (2001). Management of postpneumonectomy empyema and bronchopleural fistula. Semin Thorac Cardiovasc Surg.

[7] Ditterich D, Rexer M, Rupprecht H (2006). Vakuumtherapie beim Pleuraempyem – Erste Erfahrungen mit der Anwendung im Pleuraspalt. Zentralbl Chir.

[8] Ewig S, Kolditz M, Pletz M, Altiner A, Albrich W, Droemann D, Flick H, Gatermann S, Krüger S, Nehls W, Panning M, Rademacher J, Rohde G, Rupp J, Schaaf B, Heppner H‑J, Krause R, Ott S, Welte T, Witzenrath M. S3-Leitlinie Behandlung von erwachsenen Patienten mit ambulant erworbener Pneumonie – Update 2021. AWMF-Register-Nr. 020-020. 10.1055/a-1497-0693 34198346

[9] Ferreiro L, Porcel JM, Bielsa S, Toubes ME, Alvares-Dobano JM, Valdés L (2018). Management of pleural infections. Expert Rev Respir Med.

[10] Hecker E, Hamouri S, Müller E, Ewig S (2012). Pleural empyema and lung abscess: current treatment options. Zentralbl Chir.

[11] Höfken H, Hecker E (2013). Standadisierte Therapie des Pleuraempyems per VATS. Zentralbl Chir.

[12] Hofmann HS, Schemm R, Grosser C, Szöke T, Sziklavari Z (2012). Vacuum-assisted closure of pleural empyema without classic open-window thoracostomy. Ann Thorac Surg.

[13] Kühn F, Rau BM, Klar E, Schiffmann L (2014). Endoscopic vacuum therapy after iatrogenic oesophageal perforation—a case report. Zentralbl Chir.

[14] Kühn F, Zimmermann J, Beger N, Wirth U, Hasenhütl SM, Drefs M, Chen C, Burian M, Karcz WK, Rentsch M, Werner J, Schiergens TS (2021). Endoscopic vacuum therapy for treatment of rectal stump leakage. Surg Endosc.

[15] Loske G, Müller CT (2019). Tips and tricks for endoscopic negative pressure therapy. Chirurg.

[16] Loske G, Müller J, Röske A, Majert D, Schulze W, Mueller CT (2022). Closure of a duodenal cutaneous fistula with endoscopic negative pressure therapy using a thin open-pore film drain—an easy tool and simple method. Endoscopy.

[17] Loske G, Rucktaeschel F, Schorsch T, Moenkemueller K, Müller CT (2019). Endoscopic negative pressure therapy (ENPT) for duodenal leakage—novel repair technique using open-pore film (OFD) and polyurethane-foam drainages (OPD). Endosc Int Open.

[18] Loske G, Rucktaeschel F, Schorsch T, van Ackeren V, Stark B, Müller CT (2015). Successful endoscopic vacuum therapy with new open-pore film drainage in a case of iatrogenic duodenal perforation during ERCP. Endoscopy.

[19] Loske G, Schorsch T, Rucktaeschel F, Schulze W, Riefel B, van Ackeren V, Müller CT (2018). Open-pore film drainage (OFD): a new multipurpose tool for endoscopic negative pressure therapy (ENPT). Endosc Int Open.

[20] Matzi V, Lindenmann J, Porubsky C, Mujkic D, Maier A, Smolle-Jüttner FM (2006). V.A.C.®-Behandlung: Neue Wege im Management septischer Komplikationen der Thoraxchirurgie. Zentralbl Chir.

[21] Matzi V, Lindenmann J, Porubsky C, Neuboeck N, Maier A, Smolle-Jüttner FM (2007). Intrathoracic insertion of the VAC device in a case of pleural empyema 20 years after pneumonectomy. Ann Thorac Surg.

[22] Munguía-Canales DA, Vargas-Mendoza GK, Alvarez-Bestoff G, Calderón-Abbo MC (2013). Management of pleural empyema with a vacuum-assisted closure device and reconstruction of open thoracic window in a patient with liver cirrhosis. Arch Broncopneumol.

[23] Reichert M, Pösentrup B, Hecker A, Padberg W, Bodner J (2018). Lung decortication in phase III pleural empyema by video-assisted thoracoscopic surgery (VATS)-results of a learning curve study. J Thorac Dis.

[24] Sandhaus T, Striebitz L, Doenst T, Steinert M (2019). Behandlungsstrategien beim Pleuraempyem – Analyse eigener Daten. CHAZ.

[25] Schumpelik V, Bleese N, Mommsen U (2017). Thoraxchirurgie, Erkrankungen der Pleura, Pyothorax (Pleuraempyem). Kurzlehrbuch Chirurgie.

[26] Shen KR, Bribriesco A, Crabtree T, Denlinger C, Eby J, Eiken P, Jones DR, Keshavjee S, Maldonado F, Paul S, Kozower B (2017). The American Association for Thoracic Surgery consensus guidelines for the management of empyema. Thorac Cardiovasc Surg.

[27] Sommer K, Hüfner T, Krettek Ch (2009). Die Behandlung traumatischer Weichteilwunden mit der Vakuumtherapie – Grundlagen, Indikationen und klinische Anwendung. OP-JOURNAL.

[28] Sziklavari Z, Ried M, Hofmann HS (2015). Intrathoracic vacuum-assisted closure in the treatment of pleural empyema and lung abscess. Zentralbl Chir.

[29] Tscheliessnigg K-H, Uranüs S, Pierer G (2005). Pleuraempyem. Lehrbuch der Allgemeinen und Speziellen Chirurgie.

[30] Varker K, Ng T (2006). Management of empyema cavity with the vacuum-assisted closure device. Ann Thorac Surg.

